# Genomic DNA Enrichment Using Sequence Capture Microarrays: a Novel Approach to Discover Sequence Nucleotide Polymorphisms (SNP) in *Brassica napus* L

**DOI:** 10.1371/journal.pone.0081992

**Published:** 2013-12-03

**Authors:** Wayne E. Clarke, Isobel A. Parkin, Humberto A. Gajardo, Daniel J. Gerhardt, Erin Higgins, Christine Sidebottom, Andrew G. Sharpe, Rod J. Snowdon, Maria L. Federico, Federico L. Iniguez-Luy

**Affiliations:** 1 Saskatoon Research Centre, Agriculture and Agri-Food Canada, Saskatoon, Saskatchewan, Canada; 2 Genomics and Bioinformatics Unit, Agriaquaculture Nutritional Genomic Center (CGNA), Temuco, Louisiana, United States of America Araucanía, Chile; 3 Roche NimbleGen, Inc., Madison, Wisconsin, United States of America; 4 Plant Biotechnology Institute, National Research Council Canada, Saskatoon, Saskatchewan, Canada; 5 Department of Plant Breeding, Justus Liebig University, Giessen, Germany; University of Kentucky, United States of America

## Abstract

Targeted genomic selection methodologies, or sequence capture, allow for DNA enrichment and large-scale resequencing and characterization of natural genetic variation in species with complex genomes, such as rapeseed canola (*Brassica napus* L., AACC, 2n=38). The main goal of this project was to combine sequence capture with next generation sequencing (NGS) to discover single nucleotide polymorphisms (SNPs) in specific areas of the *B. napus* genome historically associated (via quantitative trait loci –QTL– analysis) to traits of agronomical and nutritional importance. A 2.1 million feature sequence capture platform was designed to interrogate DNA sequence variation across 47 specific genomic regions, representing 51.2 Mb of the *Brassica* A and C genomes, in ten diverse rapeseed genotypes. All ten genotypes were sequenced using the 454 Life Sciences chemistry and to assess the effect of increased sequence depth, two genotypes were also sequenced using Illumina HiSeq chemistry. As a result, 589,367 potentially useful SNPs were identified. Analysis of sequence coverage indicated a four-fold increased representation of target regions, with 57% of the filtered SNPs falling within these regions. Sixty percent of discovered SNPs corresponded to transitions while 40% were transversions. Interestingly, fifty eight percent of the SNPs were found in genic regions while 42% were found in intergenic regions. Further, a high percentage of genic SNPs was found in exons (65% and 64% for the A and C genomes, respectively). Two different genotyping assays were used to validate the discovered SNPs. Validation rates ranged from 61.5% to 84% of tested SNPs, underpinning the effectiveness of this SNP discovery approach. Most importantly, the discovered SNPs were associated with agronomically important regions of the *B. napus* genome generating a novel data resource for research and breeding this crop species.

## Introduction

*Brassica napus* L. var *oleifera* Delile (2n=4x=38, AACC) known as rape, oilseed rape or rapeseed belongs to one of the three cultivated allotetraploid *Brassica* crops that form U’s triangle [1]. Since the 1990´s, the cultivation of rapeseed has been primarily devoted to the production of edible oil, high value protein meals and more recently, biofuels [2,3]. Canadian scientists developed varieties which were declared suitable for human and animal consumption by the United States Food and Drug Administration in 1985 [4,5] after reducing the seed levels of two antinutritional factors, erucic acid (<2% of total oil) and glucosinolates (<30 mg in the meal). This transformation of rapeseed from an industrial (lubricants) into an edible oil [6,7] has been regarded as one of the major achievements of modern plant breeding [5]. In addition to improving the nutritional quality of rapeseed, major breeding efforts have been devoted to increasing seed yield (e.g. hybrid development) and identifying resistance genes for blackleg and sclerotinia, among other diseases [3,8].

Rapeseed researchers have developed a large number of genomic tools in order to accelerate the breeding process and to understand the genetic basis of complex traits in a polyploid genome [3,9]. Among these tools, the development and application of molecular markers in rapeseed research has prevailed and has grown immensely since their humble beginnings in the 1980s to the impressive high-throughput technologies of today [2,10,11]. Molecular markers like Restriction Fragment Length Polymorphism (RFLP), Randomly Amplified Polymorphic DNA (RAPD) and Simple Sequence Repeats (SSR) have at one time or another, all been developed with the ultimate goal of associating DNA sequence differences with desirable phenotypic variation [12]. Largely, this task was conducted by developing more than 34 molecular linkage maps (reviewed in 3) that were used as reference charts for quantitative trait loci (QTL) analysis [13-20]. Additionally, molecular markers have also been used to understand the structure of the *Brassica* A and C genomes [13,21-23], organize germplasm collections [24,25] and predict heterosis through the formation of heterotic groups for their subsequent combination in hybrid varieties [19,26].

The recent availability of sequence data for *Brassica* crops [2,9,27,28] is now steering researchers into developing newer, more efficient and high-throughput molecular markers. Among these, the most popular choice of marker is single nucleotide polymorphisms (SNPs), which were first embraced in human and animal genetics [29,30] and have subsequently made their way into plant genomics and genetics [30-32]. Hayward et al. [33] reviewed the latest technologies available for SNP discovery as well as the potential application that SNP markers may have in *B. napus* genetic and genomic research. 

A logical step during the process of associating molecular markers with desirable phenotypes is to make use of all the genetic and genomic information available for a given individual and/or population through targeted discovery approaches. This can be achieved by enriching for chromosomal regions underlying QTLs of agronomical and nutritional interest via hybridization-based platforms, in combination with high-throughput next generation sequencing (NGS) technologies. This could provide rapeseed researchers with a unique opportunity to discover SNPs under QTL peaks and their corresponding confidence intervals. Given the vast number of QTL mapping studies for various agronomical and nutritional traits in rapeseed [2] and the recent advances made in genomics and bioinformatics [33], it is now possible to investigate sequence variation (among individuals) of specific QTL targeted genomic regions with the aim of discovering SNPs tightly linked to a desired phenotype. This can also be useful to further elucidate the genetic and molecular basis underlying a trait. 

NGS offers a faster, more systematic and cost-effective way of assessing genetic variation [34,35]. However, a major challenge to broadly apply these NGS technologies continues to be the targeted DNA enrichment of large and complex eukaryotic genomes. For example, sequencing a thousand genes would require designing and synthesizing several thousands of PCR primers and performing several thousands of PCR reactions, a costly and lengthy process. Recently, the development of microarray-based or liquid-based genomic selection methods have proven extremely useful for the isolation of user-defined unique genomic sequences in one single enrichment step [36-38]. These methodologies are commonly referred to as “Sequence Capture” and have been broadly applied in human genetics [39-41]. In plant genetics, resequencing of targeted genomic areas has resulted in the discovery of SNP markers, the study of gene copy number variation and characterization of homoeologous coding and non-coding sequences in allopolyploid genomes [42-45]. One crucial advantage of using sequence capture protocols in complex polyploid genomes is that duplicated loci can be specifically targeted and subsequently resolved using their interlocus polymorphisms, avoiding the risk of illegitimately comparing duplicated copies in the genome [2,32]. 

In this study, we have combined the use of sequence capture with NGS technologies in order to i) target 47 genomic regions underlying traits of agronomical and nutritional interest previously identified via QTL analysis, ii) re-sequence 10 contrasting genotypes, iii) discover and validate SNP markers for the interrogated QTL regions, and iv) characterize the discovered SNPs (genic vs. intergenic; coding vs. intronic sequence; transition vs. transversion). 

## Materials and Methods

### *Brassica napus* plant material

The ten genotypes used in this study were grown under greenhouse conditions in a 16h/day photoperiod regime. These varieties or breeding lines were chosen in order to exploit available sequence, genotypic and phenotypic information (Table S1). Genotypes were classified as either low or high in both erucic acid and glucosinolates (hereinafter referred to as 00 and ++, respectively). Briefly, Tapidor (00 and late flowering) and Ningyou7 (++ and early flowering) are two divergent winter cultivars and the parents of a doubled haploid (DH) population that has been used to map several QTL for seed oil and erucic acid content [46]. Express (00 elite variety) and V8 (++ synthetic line) are the parents of another DH population that has and continues to be extensively tested for a number of useful agronomical and nutritional traits including seed yield, seedling vigour, heterosis, oil content and nitrogen use efficiency [47,48]. DH12075 (00, early flowering and blackleg resistant) and PSA12 (seedling vigor) are spring type parents of the Canadian reference mapping population [13]. Extensive genomic and EST sequence data are available from DH12075. YN-429 (early flowering) is a yellow seed line that is also the parent of a DH population with DH12075. This DH population has been genotyped and is presently being phenotyped for agronomical and quality traits (Isobel Parkin, personal communication). Rainbow (00) is an Australian spring type that has been bred for robust blackleg resistance. CGNA1 (00, late flowering and susceptible to sclerotinia) and CGNA 2 (++, early flowering and susceptible to blackleg disease) are winter type genotypes that have been bred under the agro-climatic conditions of south central Chile. 

### NGS library preparation

Nuclei DNA was extracted from flower buds and leaf tissue following standard lab protocols [49]. The DNA was checked for quality (A260/A280 ratio=1.98-2.0) and quantified using a Nanodrop spectrophotometer (10-15 ug per sample). All ten genotypes were used to prepare 454 Life Sciences NGS libraries. In addition, two lines (DH12075 and Express) underwent Illumina library preparation in order to compare the results from the two approaches. Briefly, 454 GS-FLX Titanium sequencing libraries were constructed using the 454 Life Sciences (454, Branford, CT) GSFLX Titanium Kit as described in the user guide. The entire genomic DNA product from this library preparation (e.g. SST library) was used as template in a Pre-Hybridization Linker Mediated PCR (LMPCR) reaction to ensure that the majority of the molecules contained adapters on both sides of the inserts. LMPCR consisted of 5 reactions each containing 5µl 10X Platinum High Fidelity Polymerase Buffer (Invitrogen, CA), 2.5 µl MgSO_4_, 1 µl 25nM dNTPs, 1 µl of 25 µM Primer A (5’-ACCATCTCATCCCTGCGTGTC), 1 µl of 25 µM Primer B (5'-CCTATCCCCTGTGTGCCTTG), 0.4 µl Platinum High Fidelity Polymerase (Invitrogen, CA). DNA in equal amounts was apportioned for each of the five reactions, with water to 50 µl. The reactions were then subjected to 94°C for 4 minutes followed by 8 cycles of: 94°C for 30 seconds, 1 minute at 58°C and 1.5 minutes at 68°C. The last step was an extension at 72°C for 5 minutes. The reactions were then kept at 4°C until further processing. The amplified material was recovered with a Qiagen Qiaquick column according to the manufacturer’s instructions (Qiagen, CA), except the DNA was eluted in 50 µl water instead of the EB buffer. The DNAs were quantified using the NanoDrop-1000 (Wilmington, DE) and the library was evaluated electrophoretically with an Agilent Bioanalyzer 2100 (Agilent, CA) using a DNA 7500 chip. The library fragment sizes were found to be 500 to 700 bp, which are well within the expected range.

Illumina Paired End libraries (Illumina Inc., CA) were constructed using Illumina’s PE Kit, with modifications. The agarose gel excision was performed at 250-300 base pairs (bp) to produce libraries with an approximate insert size of 130-180 bp. DNA was gel purified using a Qiaquick column (Qiagen, CA) and eluted in 30µl of water. The entire recovery product was used as template in the pre-hybridization library amplification via the Illumina sequencing adapters (i.e. LMPCR). Pre-hybridization LMPCR consisted of one reaction containing 50 µl Phusion High Fidelity PCR Master Mix (New England BioLabs, MA), 2µM of primers Illumina PE 1.0: (5'-AATGATACGGCGACCACCGAGATCTACACTCTT TCCCTACACGACGCTCTTCCGATC*T) and 2.0: (5'-CAAGCAGAAGACGGCATACGAGATCGGTCTCGGCATTCCTGCTGAACCGCTCTTCCGATC*T) where the asterisk denotes a phosphorothioate bond, 30µl DNA, and water up to 100µl. PCR cycling conditions were as follows: 98°C for 30 seconds, followed by 8 cycles of 98°C for 10 seconds, 65°C for 30 seconds, and 72°C for 30 seconds. The last step was an extension at 72°C for 5 minutes. The reaction was then kept at 4°C until further processing. The amplified material was cleaned again with a Qiagen Qiaquick column according to the manufacturer´s instructions, except the DNAs were eluted in 50 µl water. The DNAs were quantified using a NanoDrop-1000 (Thermo Scientific, DE) and the library was evaluated electrophoretically with an Agilent Bioanalyzer 2100 using a DNA1000 chip (Agilent, CA). The mean library fragment size was found to be 328 bp. Fragment sizes are well in range with the expected ones.

### Selection of *B. napus* genome sequence information to be captured

**Figure 1:**
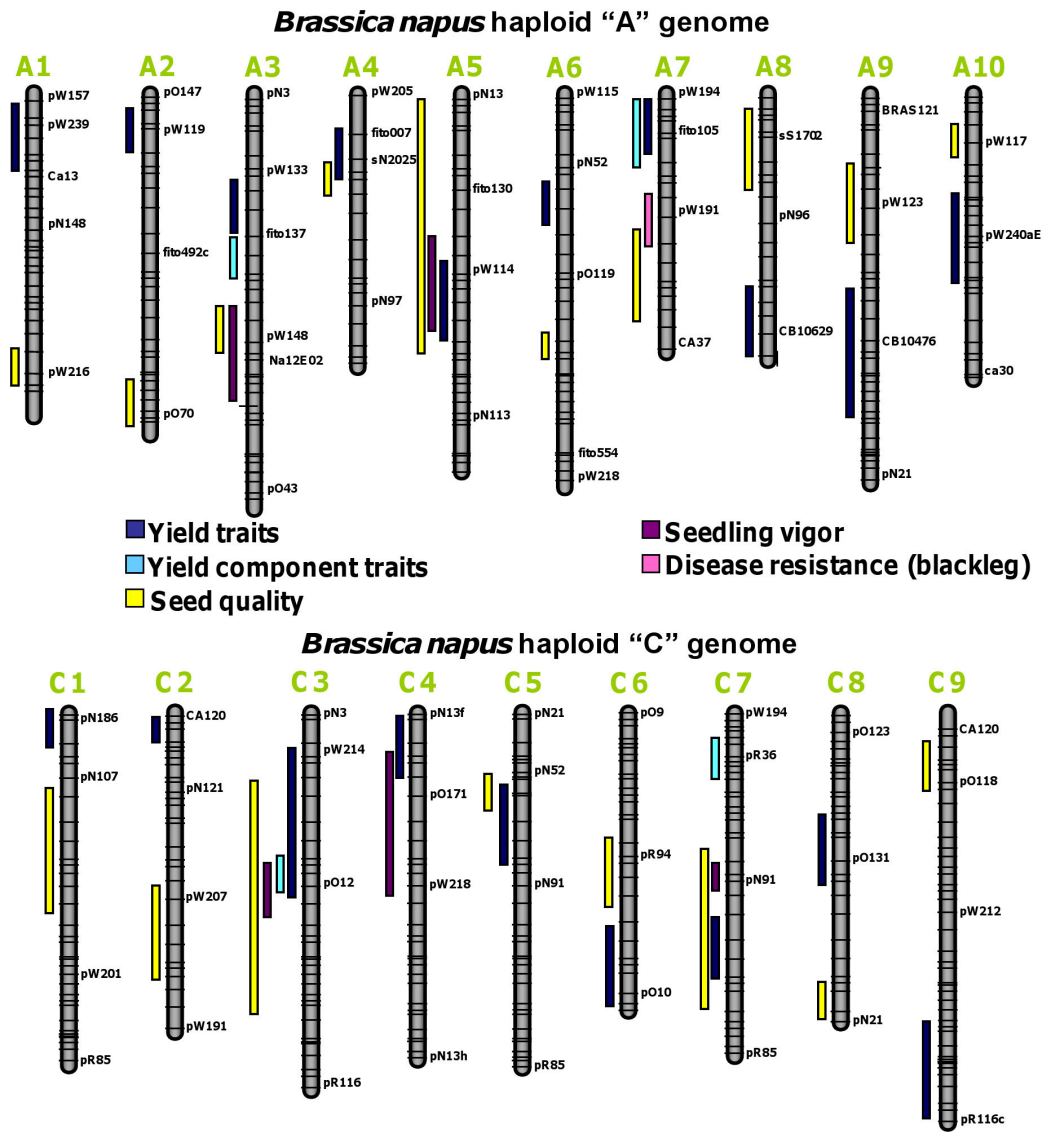
Selected genomic regions underlying traits of agronomical and nutritional interest in *B. napus*. Map integration was conducted according to common molecular markers and parental lines used in three different mapping studies [15, 22, 50]. QTL locations were inferred from relative map positions previously described [13,15,17-19, 22] always using common sets of molecular markers and genetic stocks.

A total of 47 genomic regions from the A and C genomes of *B. napus* L. that were previously found to be associated with yield and yield component traits, seedling vigor, seed quality and disease resistance traits (Figure 1) were selected to develop probes for the sequence capture platform. This was conducted by selecting sequenced molecular markers that were located under QTL peaks and identifying their corresponding intervals in the 10 A genome haploid chromosomes of *B. napus* (Table S2). Since the genome sequence for *B. rapa* (equivalent to the A genome progenitor of *B. napus*) was available, sequences were extracted from the A genome according to the coordinates/regions identified to be associated with traits of interest. Predicted genes from those regions were BLASTed against available *B. oleracea* (or C genome) contigs to identify the orthologous regions. The regions from the C genome that had the most compelling synteny with the A genome segments were selected. This information was compiled into 883 FASTA sequences (Capture Design Reference) from sequenced scaffolds of *B. rapa* [51] and preliminary contigs from *B. oleracea* (Parkin and Sharpe, unpublished data), totaling approximately 51.2 Mb (38.8 Mb and 12.4 Mb corresponding to the A and C *Brassica* genomes, respectively).

### Sequence capture design

The first step during the design of the sequence capture array was the development of a probe database set (70 to 100-mer oligos to be placed on the sequence capture microarray device) for the 51.2 Mb of selected genomic sequence. The second step involved the selection of probes using proprietary Roche NimbleGen software algorithms. As a result, a set of probes was selected to provide a 93.4% to 98.3% coverage of the targeted genomic sequence. 

### Sequence capture hybridization

Sequence capture array hybridization for all genotypes was conducted by Roche NimbleGen (Madison, WI) following their proprietary protocol. The 2.1M features array used to hybridize the ten genotypes had a coverage representation of 93.4-98.3% of the target areas of interest. The captured or enriched DNA was assessed using quantitative PCR. Five genotypes (DH12075, Ningyou_7, PSA12, Tapidor and YN-429) were captured using a solid array platform and analyzed using a 7500 Bioanalyzer chip. The remaining five genotypes (Rainbow, Express, V8, CGNA1 and CGNA2) were captured using a liquid platform and analyzed using a 1000 Bioanalyzer chip. Captured DNA elutions after hybridization and enrichment assessment were lyophilized and sent for NGS.

### NGS of enriched DNA: 454 Life Sciences and Illumina chemistries

454 Life Sciences chemistry NGS was conducted for all ten captured genotype DNAs (Table 1). In addition, captured DNA samples from DH12075 and Express underwent NGS using the Illumina HiSeq. Standard protocols described by the manufacturers (454 Life Sciences, a Roche company, CT and Illumina, Inc., CA) were used for sequencing the captured DNA at Roche Nimblegen (Madison, WI). Sequence data files were deposited in the Short Read Archive of NCBI under the BioProject ID: PRJNA212782. Sequence reads were mapped to a reference dataset as described below.

**Table 1:** Summary of Next Generation Sequencing (NGS) results.

**Lines**	**Chemistry**	**Sequenced Reads**	**Total Num. of Bases**	Avg. Length*	**SD Length**	**Avg. Q-score**	**SD Q-score**
DH12075	454	1,289,496	450,850,003	350	131.6	33.1	8.4
PSA12	454	826,680	289,078,010	350	101.1	32.6	8.5
Express	454	827,074	313,062,567	379	105.4	31.4	8.4
V8	454	711,244	261,475,787	368	121.5	33.7	8.2
Tapidor	454	778,116	257,650,369	331	95.1	31.6	8.5
Ningyou-7	454	803,553	288,413,071	359	112.8	33.2	7.9
Rainbow	454	742,283	248,197,873	334	129.9	33.7	8.1
YN-429	454	735,005	254,920,458	347	101.3	32.4	8.5
CGNA1	454	742,361	267963689	364	118.7	33.1	8.4
CGNA2	454	717,016	270,568,629	374	117.6	33.3	8.3
DH12075	Illumina	167,215,495	8,225,138,523	49	-	40	2.3
Express	Illumina	184,559,482	10,908,314,681	59	-	42	2.1

Abbreviations: Num.=Number; Avg. = Average; SD = Standard deviation; Q-score = Quality.

* Length in base pairs (bp).

### Reference sequence datasets

Three reference sequence datasets were developed to fully interrogate the capture design process. The capture design reference (883 sequences) was previously described. A second dataset containing the capture design sequences as well as their orthologous sequences was generated to increase the resolution of homoeologous sequences during the read mapping. To identify the orthologue of each sequence used in the capture design, sequences were individually aligned to the pseudomolecules of the complementary genome using NUCmer (MUMmer 3.0 package, [52]). Resulting delta files from the NUCmer alignments were passed through a Perl script to filter out the majority of the repetitive hits. The resulting files were plotted using mummerplot (MUMmer 3.0 package, [52]) and investigated manually before the complementary region was determined. The third dataset contains *de novo* assembles of the *Brassica* A and C genome pseudomolecules (representing 283.8 Mb of *B. rapa* and 488.6 Mb of *B. oleracea*, respectively) and was used to determine sequence coverage in the target and non-target regions of the genome.

### Captured DNA read mapping and SNP discovery

The sequence reads (for all ten genotypes) were aligned to the each of the three reference data sets using the CLC Bio Genomics Server software version 4.5.1 (Katrinebjerg, Denmark; www.clcbio.com). Default parameters for the mapping algorithm were used except the mapping identity parameter was increased to 98% in order to resolve homoeologous sequence reads. 

The mapped reads were then interrogated for sequence variation using the CLC Bio probabilistic variant calling tool with a minimum depth of 3 reads for the 454 data, and 8 reads for the Illumina data. Custom Perl scripts were used to compile information from SAM files and unfiltered SNP calling files generated by CLC Bio. The SNPs were filtered according to the following criteria: i) removal of SNPs with multiple variations within the 10 lines, ii) SNPs containing a high proportion of heterozygous calls or bias, and iii) SNPs without flanking sequence suitable for KASPar or Illumina Infinium design. For KASPar primer design, a SNP requires 100 bp of flanking sequence on both sides of the SNP. For Illumina Infinium assay design, a SNP requires 60 bp of flanking sequence that is free from additional SNPs on at least one side.

SNP characterization: filtered and unfiltered SNPs were characterized as genic or intergenic using the available annotation of the A and C diploid *Brassica* genomes. Coordinates for genes and the corresponding coding sequences (CDS) were extracted from GFF annotation files using a Perl script and SNP positions were then compared to the list of gene coordinates. For each of these sets the breakdown of transitions and transversions was determined using standard UNIX utilities to extract and count the SNP types. Coverage of the target and non-target regions of the sequence capture was compared using BEDTools [53] and custom Perl scripts.

### SNP Validation

KASPar chemistry validation assay: DNA was extracted from two segregating populations and a diversity panel using the CTAB procedure scaled for 1.7 ml extractions [54]. DNA was quantified using the Quant-iT PicoGreen dsDNA reagent (Invitrogen Inc., CA) and adjusted to 5 ng/µl. The KASPar assay version 4.0 (LGC Genomics, UK) was used to validate a set of 100 SNPs discovered in this study. The reactions were conducted according to the manufacturer´s instructions in an 8 µl final volume reaction. The assays were carried out in a *B. napus* diversity panel composed of 25 highly inbred lines (Rod Snowdon, personal communication) and in two segregating populations (DH12075 x PSA12 and V8 x Express) of 30 and 29 individuals, respectively (Table S3). SNPs randomly distributed in the 47 target genomic areas were chosen for KASPar validation (Table S4). Nineteen and 37 KASPar oligonucleotide sets were designed to specifically detect polymorphisms in the DH12075 x PSA12 and V8 x Express populations, respectively. In addition, 44 KASPar oligonucleotide sets were designed to detect polymorphisms in both populations at the same time. All the primer sets were designed using PrimerPicker (KBioscience, 2009) with default parameters. 

60K Illumina array validation: SNPs were assayed against a selection of *B. napus* lines using the *Brassica* 60K iSelect 24x1HD Custom Genotyping Beadchips and samples were prepared and assayed as per the Infinium HD Assay Ultra Protocol (Infinium HD Ultra User Guide 11328087_RevB, Illumina, Inc. San Diego, CA). The *Brassica* 60K beadchips were imaged using an Illumina HiScan system, and the SNP alleles were called using the Genotyping Module v1.9.4, within the GenomeStudio software suite v2011.1 (Illumina, Inc. San Diego, CA).

Mapping of a subset of validated SNP markers: linkage analysis and map construction were conducted separately for each population using JoinMap® v4.0. Linked loci were grouped using a LOD threshold of 5-8 and a maximum recombination fraction of 0.4. Grouped marker loci, including the newly mapped SNP markers, were arranged into a scoring matrix using JoinMap® v4.0. The data set was inspected for the presence of spurious double crossovers (identified using JoinMap® v4.0 data tool kit) generated by missing data and taken into account in the final linkage group construction. After the original scores were rechecked, a final linkage map was constructed for each population. Map distances in centiMorgans (cM) were calculated using the Kosambi mapping function.

## Results

### *Brassica napus* genomic DNA sequence enrichment using 2.1M NimbleGen custom sequence capture arrays

To investigate meaningful DNA sequence variation, genomic regions of interest were selected for sequence capture that had shown consistent QTL locations in *B. napus* genome across a variety of different genotypes, years and experimental locations. Examples of consistent QTL findings for important agronomical and nutritional traits (e.g. yield, yield components, seed quality, seedling vigor, disease resistance) could be found in several studies [13-19]. As a result, a total of 47 genomic regions comprising a total of 51.2 Mb (38.8 Mb and 12.4 Mb corresponding to the A and C *Brassica* genomes, respectively) were selected using sequenced molecular markers to approximate QTL locations in available genome sequence (Figure 1). Selected genomic sequences (51.2 Mb) were used to design solid and liquid sequence capture platforms, which allowed us to interrogate large portions of the *B. napus* genome per unit array and also to replicate internal controls, ensuring good coverage and depth for the sequencing of the captured genomic regions. 

### Captured *B. napus* genomic DNA sequencing, mapping and organization

A summary of the obtained NGS results for each of the 10 *B. napus* genotypes can be found in Table 1. Briefly, the average number of reads obtained for the 454 Life Sciences and Illumina HiSeq chemistries were 817,283 and 175,887,488, respectively. The average sequence read length was 382 bp using the 454 sequencer and 54 bp for the data obtained using the Illumina HiSeq sequencer (Table 1). Sequence quality parameters (Q-scores) were high for both NGS methods, with a 33 Q-score average for the 454 data and a 40 Q-score average for the Illumina data (Table 1).

Sequenced reads were mapped to two reference sets, the capture design reference (883 A and C Brassica genome reference sequences) and the A and C genome pseudomolecules (Parkin and Sharpe, unpublished data) [51], using the CLC Bio Genomics Server read mapping algorithm (Table 2). On average, 29% (236,966) of the 454 reads for each line uniquely matched the capture design reference, resulting in the calling of 831,767 putative SNPs for the ten genotypes. When the same 454 reads were mapped to the A and C genome pseudomolecules, an average of 479,421 reads (58%) uniquely matched the reference, increasing the number of putative SNP calls for the 10 genotypes to 1,549,530. For the two genotypes surveyed, on average 51% (88,438,604) of the Illumina HiSeq sequence reads uniquely matched the capture design reference while an average of 103,535,628 (59%) reads uniquely matched the A and C pseudomolecules increasing the number of putative SNP calls from 1,479,400 to 2,675,732.

**Table 2:** Result summary of sequenced reads mapped against the A and C *Brassica* genomes.

		**454 Chemistry Data**					
		**Capture design reference (883 Sequences)**			**A & C Genome Pseudomolecules (19 Sequences)**		
**Lines**	**SR**	**RM**	**RMp**	**SNPs**	**RM**	**RMp**	**SNPs**
**DH12075**	1,289,496	414,569	32	159,442	775,554	60	263,195
**PSA12**	826,680	241,867	29	78,683	497,595	60	138,398
**Express**	827,074	226,406	27	81,595	475,214	57	167,462
**V8**	711,244	190,312	27	65,744	411,708	58	136,009
**Tapidor**	778,116	230,934	30	75,238	453,606	58	140,756
**Ningyou-7**	803,553	240,828	30	89,273	480,899	60	150,945
**Rainbow**	742,283	207,465	28	67,546	432,873	58	132,747
**YN-429**	735,005	219,859	30	76,381	427,669	58	134,101
**CGNA1**	742,361	201,604	27	69,718	426,791	57	144,337
**CGNA2**	717,016	195,811	27	68,147	412,298	58	141,580
		**Illumina Chemistry Data**					
**Lines**	**SR**	**RM**	**RMp**	**SNPs**	**RM**	**RMp**	**SNPs**
**DH12075**	167,215,494	92,287,337	55	745,049	102,396,687	61	1,068,906
**Express**	184,559,482	84,589,870	46	834,391	104,674,569	57	1,606,826

Abbreviations: SR=sequenced reads, RM=reads matching the reference genome, RMp=reads mapped to reference genome in %, SNP=single nucleotide polymorphism.

### SNP discovery among ten contrasting *B. napus* genotypes

**Figure 2:**
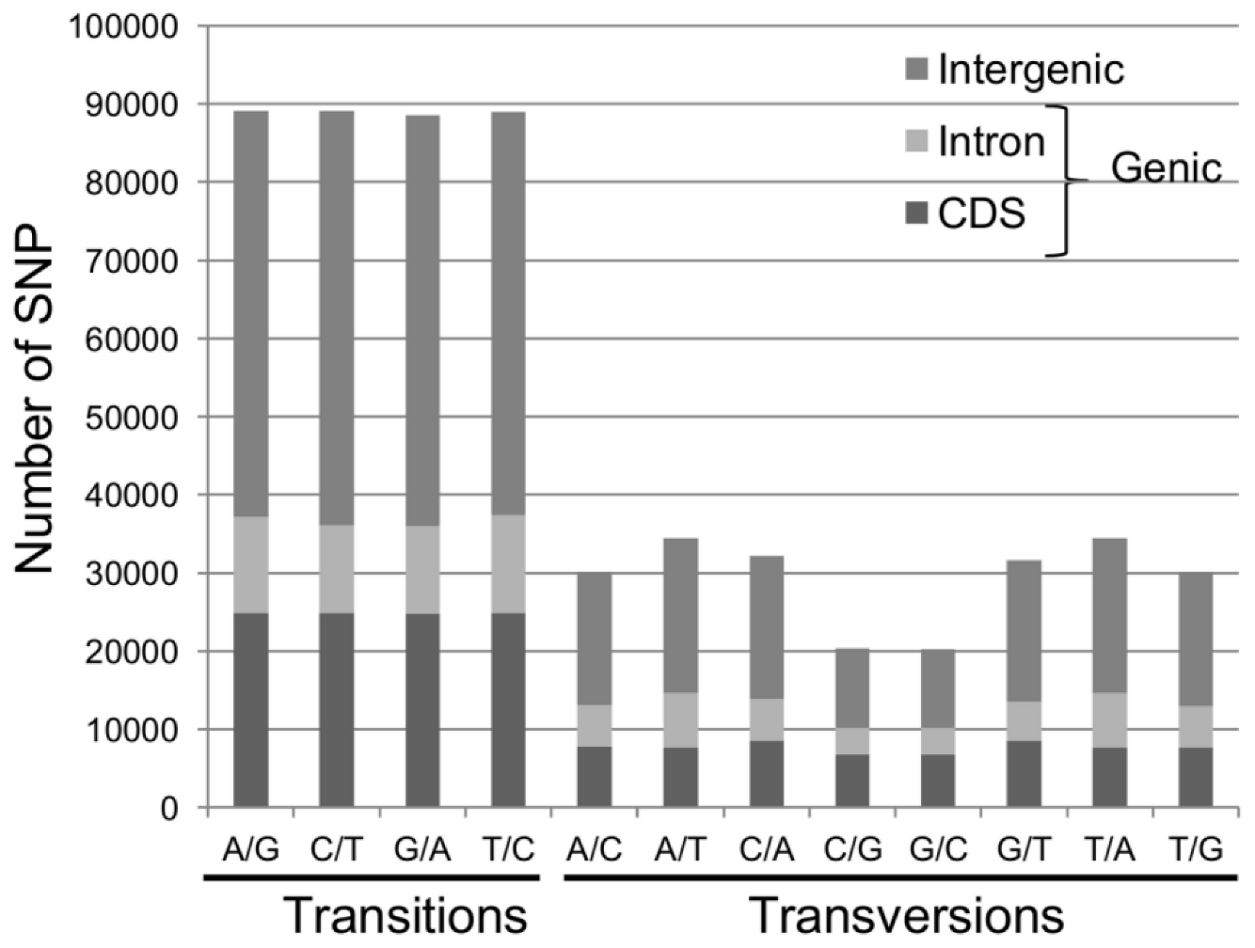
Filtered SNP types and their classification according to genomic regions. Transition and transversion SNP types were classified to show the proportion of SNPs annotated to each of three genomic regions (Intergenic, Intron, and CDS). CDS: coding sequence.

**Figure 3:**
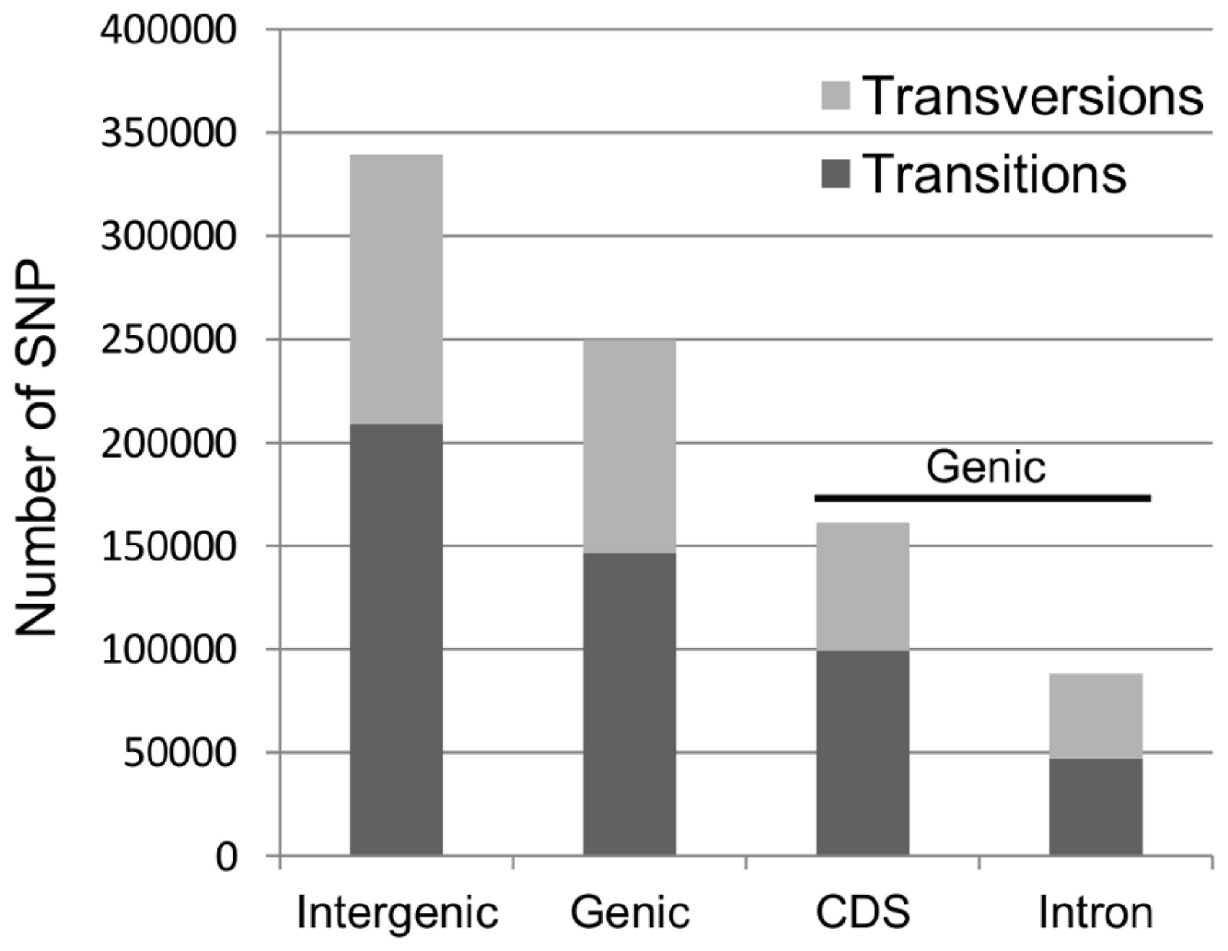
Filtered SNP counts characterization summary. Total SNP counts were classified by genomic location (Intergenic vs. Genic) and further separated into transitions and transversions. Genic SNPs are also described in terms of their location within the gene (CDS vs. intron). CDS: coding sequence.

A total of 589,367 filtered SNPs were identified from 2,740,205 unfiltered SNPs (Table 3). The discovered SNPs were classified according to type (transition vs. transversion) and whether they were found in genic or non-genic sequences. A significant number of the discovered SNPs (249,623, 42.4%) were located in a gene region and of these, 64.6% (161,189) were found within annotated coding sequences (CDS). The remaining 57.6% (339,744) were found to be intergenic. Genic and intergenic SNPs were further broken down into transitions and transversions. Transitions represent approximately 60.4% of all post-filtered SNPs (355,714), with 41.2% (146,644) of these being located in genic regions. The breakdown of each SNP type (e.g. A-T, C-A, etc.) by genomic location is illustrated in Figure 2. Of the 146,644 genic transitions, 67.9% were found in CDS, whereas 59.8% of the 102,979 genic transversions were found in CDS (Figure 3). Figures S1 and Figure S2 show these breakdowns based on a SNP set filtered only for multiple variant SNPs (minimally filtered set).

**Table 3:** Summary of SNP filtering criteria and discovery pipeline results.

**Filtering Criteria**	**Number of SNPs Excluded**	**Number of SNPs Remaining**
**None**	0	2,740,205
Multiple Variants hits	33,917	2,706,288
Heterozygous and Bias*	2,111,439	594,849
Flanking Sequence**	5,482	589,367
**Candidate SNPs**		**589,367**

* Removal of SNPs containing only heterozygous and bias SNP calls. It also removes SNPs with a percentage of heterozygous calls over a threshold (0.2).

** Removal of SNPs not meeting KASPar or Illumina Infinium flanking sequence requirements.

To assess the difference in sequencing platforms, the number of filtered SNPs unique to each sequencing type was determined for DH12075 and Express. Of the 589,367 filtered SNPs, 487,537 have a call in DH12075 or Express. Of these, 95,827 were unique to the Illumina sequencing data while 4,070 were unique to the 454 Life Sciences sequencing data. The flanking sequence for the filtered SNPs was categorized based on the downstream assay type, as KASPar, Illumina Infinium, or both. Our analysis showed that 587,261 SNPs are valid for KASPar assays, with 297,802 of those also having potential value for Illumina Infinium assay design. There were 289,459 SNPs that could only be used for KASPar design while 2,106 SNPs were only useful for Illumina Infinium design. This difference is largely due to the requirement that there are no flanking SNPs on at least one side of the target SNP for Infinium design.

### Analysis of sequence capture coverage

In order to analyze the efficacy of the sequence capture process, the depth of coverage was surveyed for both target and non-target regions. The original capture design was based on incomplete diploid *Brassica* genome sequence with a bias to the A genome (75.7% of the capture sequence). However, the strong sequence identity between the A and C genomes (on average 94%) would not allow *B. napus* homoeologous sequences to be differentiated during the capture process [55]. Thus, to accurately assess the true target region, it was necessary to identify the orthologous sequence from the complementary genome for each of the 883 sequences used in the design process. As described in the methods each sequence was aligned to the pseudomolecules of the complementary genome using NucMER, which resulted in 1,074 orthologous sequences (or 131.1 Mb) being identified, 39 for the 35 A genome capture regions and 1035 for the 848 C genome capture regions.

**Figure 4:**
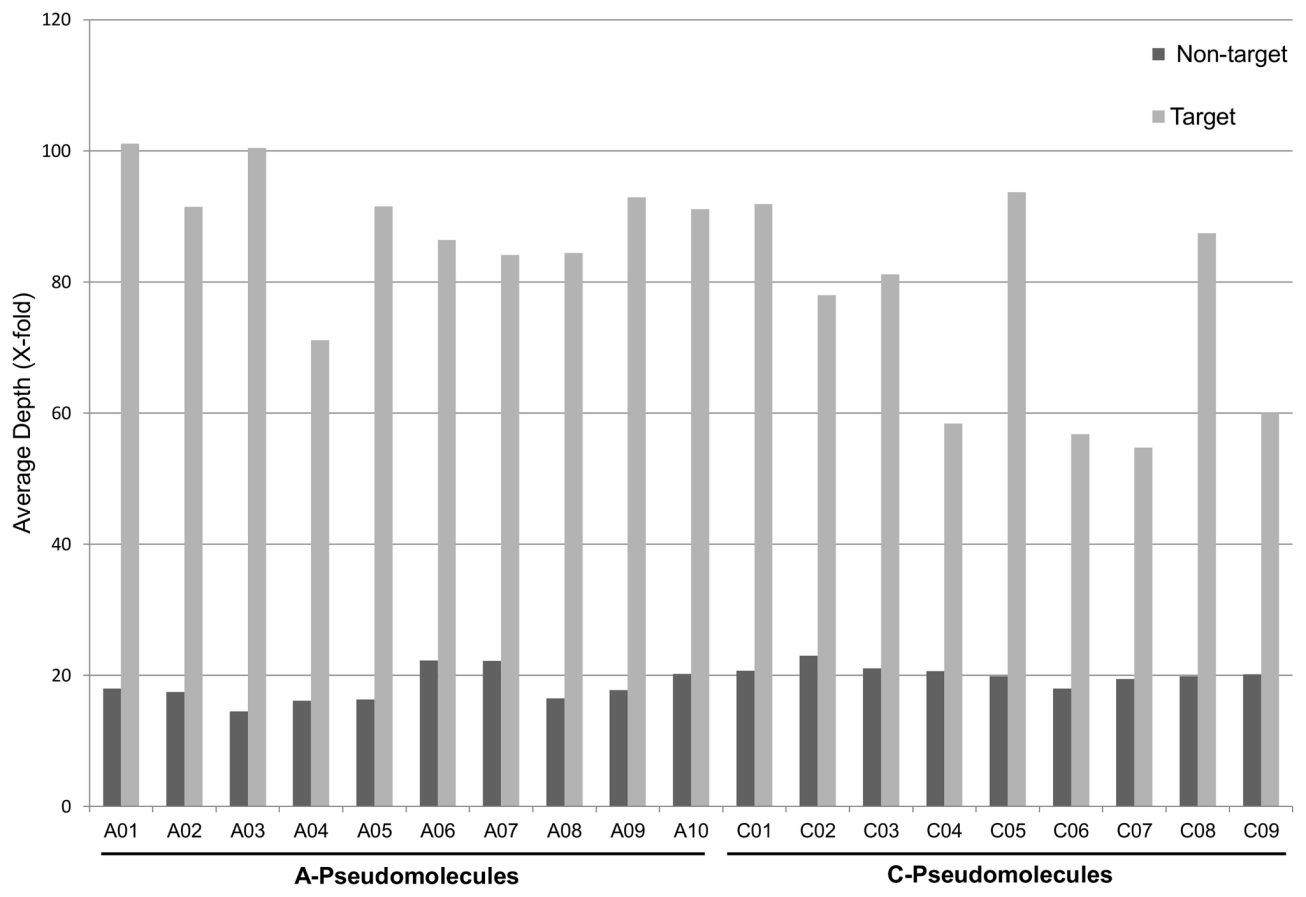
Summary of DNA sequence coverage. The average depth of coverage in captured and non-captured regions across all 19 A and C Brassica pseudomolecules is illustrated. Captured regions are those from the original sequence capture selection combined with the orthologous sequence from the complementary genome.

Sequence reads from each of the ten *B. napus* genotypes were then mapped using CLC Bio Genomics Server to three different reference sets: the original 883 capture sequences, the complemented capture regions (883 capture sequences + 1,074 orthologous sequences), and the A and C genome pseudomolecules. Sequence read mapping is presented across all ten lines for both sequencing platforms for these three reference sequence sets in Table 4. A percentage increase of reads mapped over the previous set (RMpi) is also included to illustrate better resolution of homoeologous sequences as well as the benefit of mapping to complete genome sequences. A coverage map was generated for each of the *B. napus* lines providing an average depth of coverage for target and non-target regions (Figure 4). The average depth of coverage for non-target regions was 19.6X while the average depth for the target regions was almost four-fold higher (77X). Additionally, the set of filtered SNPs were analyzed to determine their origin, 333,379 SNPs were found in the target regions, representing 56.6% of all filtered SNPs.

**Table 4:** Summary of sequence read mapping performance using multiple reference sequence sets.

		**454 Chemistry Data**					
		**Capture Design Reference (883 Sequences) **		**Capture Design with Orthologues (883 + 1074 Sequences)**		**A & C Genome Pseudomolecules (19 Sequences)**	
**Lines**	**SR**	**RM**	**RMpi**	**RM**	**RMpi**	**RM**	**RMpi**
DH12075	1,289,496	414,569	0	457,448	10.3	775,554	69.5
PSA12	826,680	241,867	0	279,959	15.7	497,595	77.7
Express	827,074	226,406	0	271,137	19.8	475,214	75.3
V8	711,244	190,312	0	230,314	21	411,708	78.8
Tapidor	778,116	230,934	0	266,100	15.2	453,606	70.5
Ningyou	803,553	240,828	0	277,338	15.2	480,899	73.4
Rainbow	742,283	207,465	0	248,757	19.9	432,873	74
YN-429	735,005	219,859	0	247,101	12.4	427,669	73.1
CGNA1	742,361	201,604	0	241,700	19.9	426,791	76.6
CGNA2	717,016	195,811	0	233,568	19.3	412,298	76.5
		**Illumina Chemistry Data**					
**Lines**	**SR**	**RM**	**RMpi**	**RM**	**RMpi**	**RM**	**RMpi**
DH12075	167,215,494	92,287,337	0	74,985,743	-18.7	102,396,687	36.6
Express	184,559,482	84,589,870	0	78,787,995	-6.9	104,674,569	32.9

Abbreviations: SR = sequenced reads, RM = reads matching the reference genome, RMpi = percentage increase in reads mapped over previous reference.

### Discovered SNP validation assays

The robustness of the SNP discovery pipeline was assessed by validating a set of 100 SNPs in two reference mapping populations (DH12075 x PSA12; Express x V8) and a *B. napus* diversity panel using the KASPar SNP assay (Table 5). Nineteen sets of KASPar oligonucleotides were designed to test for SNPs identified between the parental lines of the spring type mapping population (DH12075 x PSA12), 37 were designed to test for SNPs identified between the parental lines of the winter type mapping population (Express x V8) and 44 evaluated SNPs were common to both populations. Table 5 summarizes the results obtained using the singleplex KASPar SNP validation assay for these 100 SNPs. The assay for SNP robustness among the set of 25 diverse *B. napus* lines showed that 71% of SNPs (71/100) were informative and could discriminate among the different alleles tested. Higher rates of positively confirmed polymorphisms were observed when SNPs were designed for specific parental sets, 84% for DH12075 x PSA12 and 78% for Express x V8. When KASPar oligonucleotides were designed to test for polymorphisms in both mapping populations, the percentage of positively confirmed polymorphisms dropped to 55% probably due to the cumulative effect of failures in both populations (Table 5).

**Table 5:** Singleplex KASPar SNP validation assay of a set of 100 discovered SNP markers.

		Parental lines specific SNP*		
	Diversity Set	DH12075 x PSA12	V8 x Express	Both populations
**Individuals tested**	25	30	29	-
Total SNP tested**	100	19	37	44
**Amplification type**				
No amplification	12	0	4	8
Monomorphic	9	1	5	6
Multiple Loci	8	2	0	6
Polymorphic***	71	16	28	24
% PA	88	100	89	82
**% PS**	**71**	**84**	**78**	**55**

Abbreviations: PA = Positive Amplification; PS = Polymorphic SNPs.

* Represents markers specifically designed from SNPs that showed polymorphism for a specific set of reference mapping parental lines. For instance, polymorphisms between the spring type parents, (DH12075 and PSA12); polymorphisms between the winter type parents, (V8 and Express); and polymorphisms detected for both sets of parental lines at the same SNP locus.

** Out of the 100 SNPs tested 73 SNPs were A genome-specific and 27 were C genome-specific.

*** Polymorphic SNPs also include two dominant (presence/absence of the tested allele) marker types.

Additionally, 4,333 of the filtered SNPs were matched to SNPs used on a *Brassica* 60K Illumina Infinium array (Clarke et al, unpublished data). During array validation, tests using almost 100 *B. napus* lines indicated that 363 of the 4,333 SNPs failed, while 2,664 (61.5%) of the remaining SNPs were annotated as high quality, based on good cluster separation (AA θ 0 – 0.2 and BB θ 0.8 - 1) and a low proportion of heterozygous calls (AB Frequency <= 0.15) (data not shown). These preliminary results coupled with the KASPar assays serve as a confirmation of the filtering criteria used in the SNP discovery pipeline, suggesting sequence capture coupled with NGS is a promising tool for specific SNP marker development in polyploid genomes.

In order to confirm the chromosomal location for the discovered SNPs and associate them with the target QTL regions, we mapped a subset of the 100 validated SNP markers in both mapping populations. We were able to map 45 out of 48 SNP markers (93.8%) to the target QTL regions in the DH12075 x PSA12 mapping population. Similarly, 46 out of 50 SNP markers (92%) mapped to the target QTL regions in the Express x V8 mapping population (Figure S3). 

## Discussion

SNP discovery and molecular marker development in *B. napus* has increased remarkably during the past years since the first published work on genome-wide SNP identification by Trick and coauthors [56]. Such an increment has been mainly the result of the implementation of NGS technologies, microarrays and bioinformatics [2,57,58]. A major challenge to broadly apply NGS technologies, however, continues to be the targeted DNA enrichment of large and complex eukaryotic genomes, such as rapeseed canola (AACC, 2n=38). Sequence capture has addressed this limitation allowing for the isolation of user-defined genomic sequences in one simple step [36-38]. In this context, given the vast history of QTL mapping for various agronomical and nutritional traits in *B. napus* [13-19,48] the opportunity arose to investigate sequence variation of specific QTL targeted genomic regions [3,32]. We utilized sequence capture to target and re-sequence meta-QTL regions [2] for key complex traits including yield, yield components, seedling vigor, seed quality and disease (Table S2) in ten founder *B. napus* genotypes (Table 1) [13,47,48]. In total, 47 genomic regions were targeted comprising 51.2 Mb or approximately 4.3% of the estimated 1.2 Gbp *B. napus* genome [59]. However, since the capture process is unlikely to be able to differentiate the closely related sequences of the A and C genomes, a more accurate estimate of the genome surveyed would be 10.9% (883 + 1,074 orthologous sequences, 131.1 Mb).

Captured DNAs for the ten founder genotypes were sequenced using 454 sequencing technology. Two different reference sets were used to map the sequenced reads obtained, the capture design reference and the A and C genome pseudomolecules. This proved to be crucial allowing us to discern more efficiently between A and C homoeologues and resulted in a higher percentage (RMp) of sequence reads mapped (Table 2). The use of two different reference sets illustrated the benefit of a more complete genome reference as well as differences in sequencing platforms. Mapping of the sequenced reads obtained using Illumina HiSeq technology (2 genotypes) showed a higher RMp for the capture design reference set compared to 454. However, due to the lower specificity of the shorter reads a more subtle increase was observed on the RMp values when the Illumina sequenced reads were uniquely mapped to the A and C genome pseudomolecules, since the short reads have more opportunity for multiple equivalent matches (Table 2, Table 4). Nonetheless, the number of putative SNP calls increased for both sequencing platforms when the larger reference genome set was utilized (Table 2). In addition, the use of more complete reference sequences in the SNP identification process should increase the likelihood of identifying single loci in the amphidiploid genome. In previous attempts to use NGS to discover SNPs in *B. napus* in the absence of reference genomic sequence, the close homology between the A and C genomes often precluded the discrimination of homoeologous loci. In Trick et al [56], 87.5-91.2% of the identified genic SNPs were so called hemi-SNPs, where assays designed to such SNPs would amplify two loci, limiting their downstream application.

SNP discovery among the ten contrasting *B. napus* genotypes was performed following a stringent filtering procedure in order to remove SNPs exhibiting multiple variants at a single position, those which could result from co-assembly of homoeologous sequences and those that could represent polymorphisms that distinguish the *B. napus* species from the parental diploid species used as reference. In addition, SNPs that did not have flanking sequence suitable for either KASPar or Illumina Infinium validation were removed. As a result, 589,367 SNPs were identified from a total of 2,740,205 unfiltered SNPs (Table 3). The polymorphism rate was compared across all 10 genotypes using the equivalent 454 data sets. On average, one filtered SNP was detected every 421.3 bp in the capture regions, which is quite similar to the rate of one every 446 bp observed by Bus et al [60]. Outside of the capture regions, the rate dropped to an average of one SNP every 697.8 bp, indicating the efficiency of the capture process. Out of the filtered SNPs, 46% (268,944) mapped to the A genome pseudomolecules and 54% (320,423) mapped to the C genome pseudomolecules. Previously any bias in observed polymorphism in *B. napus* has been towards the A genome, which has been suggested to result from the introgression of *B. rapa* alleles during breeding [6,8]. However, in the current study the use of one resynthesized line (PSA12) and two *B. napus* lines with resynthesis in their recent pedigree (V8 and Ningyou) may have improved the levels of polymorphism found in the C genome.

Recently, Li and coworkers [61] explored the relative contributions of genic and nongenic SNPs to phenotypic variation for five quantitative traits in maize. Even though genic or nongenic trait associated SNPs (TASs) were found to contribute to nearly half of the phenotypic variation explained by all TASs, non-genic SNPs were significantly overrepresented among TASs [61]. This highlights the importance of discovering SNPs not only in genic but also in non-genic regions for genome wide association studies (GWAS) in crop species with complex genomes, like maize or rapeseed canola. This study provides a large and unique source of nongenic (339,744) and intronic (88,434) SNPs (Figure 3) that could not be discovered by EST-based or RNA-seq methods previously utilized [62-64]. Undoubtedly, these SNPs are not only the starting point to explore sequence polymorphisms under targeted QTL areas of the genome, but also they can be used together with other SNP sets to perform GWAS in rapeseed canola. This resource will provide a further tool to help enhance our understanding of the genetic architecture of quantitative traits in this species. 

Discovered SNPs were also classified into transitions and transversions (Figures 2 and 3). As seen in plants and other organisms the percentage of transitions (60.4%) was higher than that of transversions (39.6%) [60,65-68]. The number of the four different types of transitions was found to be balanced, whereas the number of the eight different types of transversions varied (Figure 2). A similar result was recently reported for *B. napus* using RAD sequencing [60]. The ratio of total transitions/transversions was found to be 1.52 (355,714/233,653). This ratio is in agreement with previous reports in *Brassica* where transitions/transversions ranged from 1.03 to 1.65 [60,66]. The lowest ratio of transitions/transversions (1.14; 47,065/41,369) was found in intronic sequences and the highest in CDS or exonic sequences (1.62; 99,579/61,610). The same trend between exonic and intronic regions was also observed in *B. rapa* [66]. Since transversion substitutions are more likely to produce missense and nonsense mutations, a high ratio of transitions/transversions in exonic sequences could be indicative of the presence of purifying selection on amino acid substitutions as seen in *B. rapa* [66]. 

This study combined sequence capture and NGS technologies to discover SNPs in specific areas (target) of the *B. napus* genome (QTLs). The efficiency of target sequence enrichment was evaluated by comparing the mapping of the obtained sequenced reads to multiple reference sets (Table 4). As observed in other studies [36,42], approximately 30% of the sequence reads mapped to the capture regions for the ten genotypes re-sequenced using the 454 sequencing platform. However, due to the strong homology between the A and C genomes the reference set including the complemented capture regions is perhaps a more realistic representation of the target regions, which increased the efficacy of matching for the 454 reads to approximately 35% (Table 4). Beside those regions which are still unrepresented in the reference, the requirement for unique matches in a complex polyploid genome will necessarily limit the ability to map all sequenced reads. Comparing the reads mapped to the target regions to those mapped to the A and C genome pseudomolecules indicated that on average 57.4% of the reads which could be mapped were found in the target regions. In addition, the depth of coverage which effectively dictates the ability to accurately call SNPs was surveyed for both target and non-target regions across the A and C *Brassica* pseudomolecules (Figure 4). The four-fold increase in depth of reads mapped to the target regions ensured that almost 60% of the annotated SNPs were found in these regions.

Validation of discovered SNPs is a crucial step to estimate the percentage that could be converted into robust and informative molecular markers [69]. Filtered SNPs were classified based on their available flanking sequence, and thus, the possibility of being utilized in different downstream assays as KASPar (289,459), Illumina Infinium (2,106), or both (297,802). We validated a set of 100 SNPs in two reference mapping populations (DH12075 x PSA12; Express x V8) and a *B. napus* diversity panel using the KASPar SNP assay (Table 5). Validation rates varied depending on whether the KASPar oligonucleotides were specifically designed to test for SNPs identified between the parental lines of the two mapping populations. Importantly, 71 of the 100 SNPs were also informative when tested using a *B. napus* diversity set composed of 25 lines (Table 5). Since the singleplex KASPar SNP validation assay relies on consistent template sequences, amplification failure could result from non-specific oligonucleotide annealing and not be related to the nature of the tested SNP. Therefore, corrected validation rates, not considering the 12 evaluated SNPs showing no amplification, were as follows: 81% (71/88) for the diversity set, 84 % (16/19) for DH12075 x PSA12, 85% (28/33) for Express x V8, and 67% (24/36) for both mapping populations. These rates demonstrate the robustness of our SNP discovery pipeline protocol. In addition, an independent assay evaluated 4,333 SNPs in 96 *B. napus* lines using a *Brassica* 60K Illumina Infinium array (Clarke et al, unpublished data), resulting in 61.5% validation rate. To further corroborate that the discovered SNPs correspond to polymorphisms in the target QTL regions, we mapped a subset of the 100 KASPar SNP markers in both mapping populations (Figure S3). As a result, more than 90% of the tested SNPs were confirmed to reside in the target QTL regions or its vicinity, with only two SNPs (one on each mapping population) failing to be mapped. This was probably due to the small mapping population size used to perform the linkage analysis. 

A previous study on *B napus* genome-wide SNP discovery used a combination of NGS and RAD sequencing and surveyed about 1% of the *B. napus* genome, detecting more than 20,000 SNPs among 8 inbred lines, with 84% (26/31) of the SNPs being confirmed by re-sequencing; however, none were tested in SNP assays [60]. Significantly, this approach utilized an improved reference sequence set, by combining a 95K *Brassica* unigene dataset [70] with the *B. rapa* reference sequence [27]. Our work expands on previous studies by: (i) targeting specific QTL regions representing 10.9% of the *B. napus* genome; (ii) using a more representative genomic reference, (iii) detecting more than 500 thousand SNPs among 10 contrasting genotypes, including genic (42%, 249,623) and intergenic (58%, 339,744) SNPs with an average SNP assay validation rate of over 80%. The development of high densities of robust molecular markers (e.g. SNPs) will be very powerful towards predicting the best individuals in plant breeding schemes through the use of genomic selection [71]. Illumination of the genome sequences and species-wide genetic diversity of *B. napus* crops (spring and winter types) will help to elucidate the genetic background of complex traits and broaden gene pools as a basis for more successful breeding.

## Supporting Information

Figure S1 **Minimally filtered SNP types and their classification according to genomic regions.** Transition and transversion SNP types were classified to show the proportion of SNPs annotated to each of three genomic regions (Intergenic, Intron, and CDS). In the minimally filtered SNP set, only positions with more than one variant call were removed.
(TIFF) (TIF)

Figure S2 **Minimally filtered SNP counts characterization summary.** Total SNP counts were classified by genomic location (Intergenic vs. Genic) and further separated into transitions and transversions. Genic SNPs are also described in terms of their location within the gene (CDS vs. intron). In the minimally filtered SNP set, only positions with more than one variant call were removed. CDS: coding sequence.
(TIFF) (TIF)

Figure S3 **Linkage mapping of polymorphic SNP markers discovered using sequence capture in *B. napus*.** Mapped SNP markers are illustrated in red, each horizontal bar represents a molecular marker. Linkage analysis and map position was conducted separately for each population using JoinMap® v4.0. Linked loci were grouped using a LOD threshold of 5-8 and a maximum recombination fraction of 0.4. Grouped SNP marker loci were arranged into a scoring matrix using MSExcel. Distances were assigned in centiMorgans (cM) using the Kosambi mapping function. Scoring matrixes are available upon request.
(TIFF) (TIF)

Table S1 **Genotype descriptions, fragment size estimation of captured DNA and final sample concentrations.** (DOC).
(DOCX)

Table S2 ***Brassica* A Genome Sequence Capture Coordinates.** (XLS).
(XLSX)

Table S3 **Genotypes tested in KasPar Assay.** (XLS).
(XLSX)

Table S4 **List of 100 SNP and oligonucleotides used in KasPar validation assays.** (XLS).
(XLSX)
