# Correction: Genomic DNA Enrichment Using Sequence Capture Microarrays: a Novel Approach to Discover Sequence Nucleotide Polymorphisms (SNP) in *Brassica napus* L

**DOI:** 10.1371/annotation/f92c4ed2-b4f7-47aa-97b6-75bce39dcb0e

**Published:** 2013-12-17

**Authors:** Wayne E. Clarke, Isobel A. Parkin, Humberto A. Gajardo, Daniel J. Gerhardt, Erin Higgins, Christine Sidebottom, Andrew G. Sharpe, Rod J. Snowdon, Maria L. Federico, Federico L. Iniguez-Luy

Affiliation 2 for the third, ninth and last authors is incorrect. The correct affiliation is: Genomics and Bioinformatics Unit, Agriaquaculture Nutritional Genomic Center (CGNA), Temuco, La Araucanía, Chile. 

