# Supplementary figures and images for: Genomic DNA Enrichment Using Sequence Capture Microarrays: a Novel Approach to Discover Sequence Nucleotide Polymorphisms (SNP) in Brassica napus L
Source: PLoS One. 2013 Dec 3;8(12):e81992. doi: 10.1371/journal.pone.0081992 (PMC3849492; doi:10.1371/journal.pone.0081992)

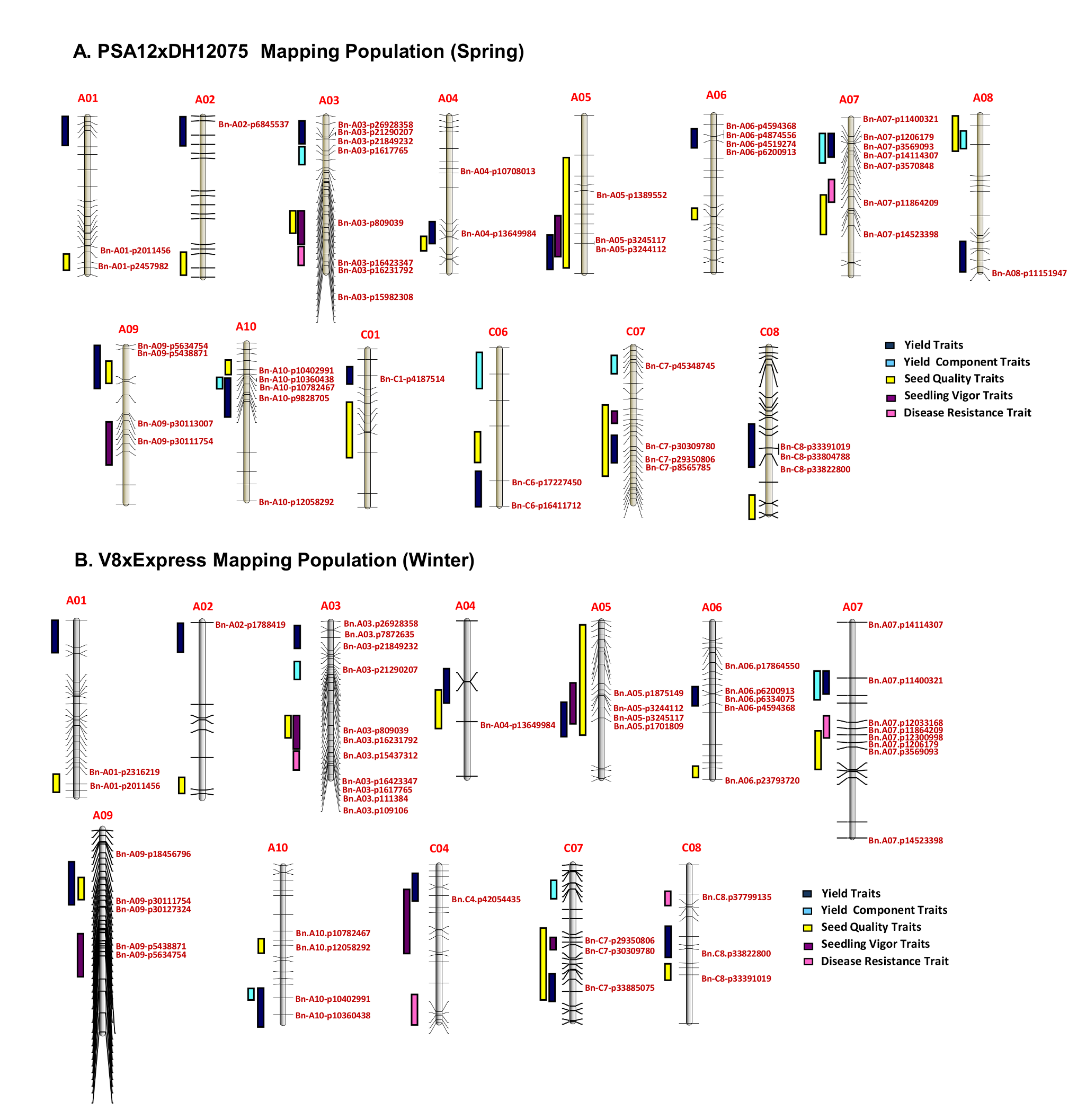

Supplement: Figure S3 — Linkage mapping of polymorphic SNP markers discovered using sequence capture in B. napus. Mapped SNP markers are illustrated in red, each horizontal bar represents a molecular marker. Linkage analysis and map position was conducted separately for each population using JoinMap® v4.0. Linked loci were grouped using a LOD threshold of 5-8 and a maximum recombination fraction of 0.4. Grouped SNP marker loci were arranged into a scoring matrix using MSExcel. Distances were assigned in centiMorgans (cM) using the Kosambi mapping function. Scoring matrixes are available upon request. (TIFF) [file pone.0081992.s003.tiff]
