# Supplementary material for: Genomic DNA Enrichment Using Sequence Capture Microarrays: a Novel Approach to Discover Sequence Nucleotide Polymorphisms (SNP) in Brassica napus L
Source: PLoS One. 2013 Dec 3;8(12):e81992. doi: 10.1371/journal.pone.0081992 (PMC3849492; doi:10.1371/journal.pone.0081992)
Supplement: Table S1 — Genotype descriptions, fragment size estimation of captured DNA and final sample concentrations. (DOC). (DOCX) [file pone.0081992.s004.docx]

**Table S1.** Genotype descriptions, fragment size estimation of captured DNA and final sample concentrations.

| **Line Name** | **Line Descriptions** | **Chemistry** | **Fragment Size (bp)** | **Concentration (ng/µl)** |
| --- | --- | --- | --- | --- |
| DH12075 | Spring type, BL R, 00, EM | 454 | 550 | 554 |
| Ningyou7 | Winter type, EM, ++ | 454 | 550 | 495 |
| PSA12 | Spring type, ST, SV | 454 | 550 | 514 |
| Tapidor | Winter type, LM, 00 | 454 | 550 | 471 |
| YN-429 | Spring type, YS, EM | 454 | 550 | 439 |
| Rainbow | Spring type, BL R, 00 | 454 | 600 | 555 |
| CGNA1 | Winter type, LM, Sc S, 00 | 454 | 630 | 58 |
| CGNA2 | Winter type, EM, BL S, ++ | 454 | 600 | 649 |
| V8 | Winter type, Resyn, ++ | 454 | 620 | 675 |
| Express | Winter type, Elite^1^, 00 | 454 | 630 | 503 |
| DH12075 | Spring type, BL R, 00, EM | Illumina | 328 | n.d. |
| Express | Winter type, Elite^1^, 00 | Illumina | 328 | n.d. |

Abbreviations: BL R=blackleg resistance; BL S=blackleg susceptible; 00=low erucic acid and low glucosinolates; ++=high erucic acid and high glucosinolates; EM=early maturing; LM=late maturing; ST=valuable seedling trait; SV=valuable seedling vigor; YS=yellow seed; Sc S=Sclerotinia susceptible; Resyn=diverse resynthesized;

^1^ Elite refer to a standard high yielding commercial or advance variety and/or parental line.
